# Role of Dietary Factors, Food Habits, and Lifestyle in Childhood Obesity Development: A Position Paper From the European Society for Paediatric Gastroenterology, Hepatology and Nutrition Committee on Nutrition

**DOI:** 10.1097/MPG.0000000000003075

**Published:** 2021-02-22

**Authors:** Elvira Verduci, Jiri Bronsky, Nicholas Embleton, Konstantinos Gerasimidis, Flavia Indrio, Jutta Köglmeier, Barbara de Koning, Alexandre Lapillonne, Sissel Jennifer Moltu, Lorenzo Norsa, Magnus Domellöf

**Affiliations:** 1 ∗ Department of Health Sciences; 2 † Department of Pediatrics, Vittore Buzzi Children's’ Hospital-University of Milan, Italy; 3 ‡ Department of Paediatrics, University Hospital Motol, Prague, Czech Republic; 4 § Newcastle Neonatal Service, Newcastle Hospitals NHS Trust and Newcastle University, Newcastle upon Tyne; 5 || Human Nutrition, School of Medicine, Dentistry and Nursing, University of Glasgow, New Lister Building, Glasgow Royal Infirmary, Glasgow, United Kingdom; 6 ¶ Dipartimento di Scienze Mediche e Chirurgiche, University of Foggia, Italy; 7 # Department of paediatric Gastroenterology, Great Ormond Street Hospital for Children NHS Foundation Trust, London, United Kingdom; 8 ∗∗ Paediatric Gastroenterology, Erasmus MC–Sophia Children's Hospital, Rotterdam, the Netherlands; 9 †† Paris Descartes University, APHP Necker-Enfants Malades hospital, Paris, France; 10 ‡‡ CNRC, Baylor College of Medicine, Houston, TX; 11 §§ Department of Neonatal Intensive Care, Oslo University Hospital, Norway; 12 |||| Pediatric Hepatology Gastroenterology and Transplantation, ASST Papa Giovanni XXIIII, Bergamo, Italy; 13 ¶¶ Department of Clinical Sciences, Pediatrics, Umeå University, Umeå, Sweden.

**Keywords:** childhood obesity, diet macronutrient intakes, early nutrition, parenting style, prevention

## Abstract

Childhood obesity has high societal and economic impact but current treatment approaches are sub-optimal. In the last decade, important studies have been conducted aiming to identify strategies to prevent obesity during critical periods of life. Updated recommendations for childhood obesity prevention are needed. We present data from systematic reviews and meta- analysis, randomised controlled trials (RCTs) and large observational studies, published from 2011 onwards that consider the possible role of the following factors in obesity development: breast-feeding; macronutrient composition and method of complementary feeding; parenting style; dietary patterns; sugar-sweetened beverage consumption; eating behaviour (eg, skipping breakfast, family dinners. etc); meal frequency and composition (fast foods, snacking), portion size; dietary modulators of gut microbiota (including pre-, pro-, and synbiotics); physical activity and sedentary behaviour. We used the Medline database and the Cochrane Library to search for relevant publications. Important research gaps were also identified. This position paper provides recommendations on dietary factors, food habits, and lifestyle to prevent childhood obesity development, based on the available literature and expert opinion. Clinical research and high-quality trials are urgently needed to resolve numerous areas of uncertainty.

## Abstract 2

Supplemental Digital Content is available in the text

**What Is Known/What Is New**

**What Is Known**

- Childhood obesity is the most prevalent food-based disorder among children and adolescents worldwide.
- Although national and international surveys report a levelling-off of the prevalence of obesity in some countries, the burden of pediatric obesity is still high, not least in Mediterranean area countries.
- The aetiology of obesity is complex, with several risk factors and mechanisms that are interconnected.

**What Is New**

- The European Society for Paediatric Gastroenterology, Hepatology, and Nutrition Committee on Nutrition provides an update of the previous position paper (2011) that also evaluates the role of dietary patterns and food habits in the prevention of obesity in children and adolescents ages 2 to 18 years. Factors in infants and children <2 years of life, as well as the role of physical activity and sedentary behaviour will also be considered in this updated version.
- A logic model was developed to demonstrate aspects of the complex system underlying the development of child and adolescent obesity.
- Updated recommendations are provided to prevent childhood obesity.

## INTRODUCTION

Obesity is a global public health problem associated with a wide range of metabolic abnormalities and a negative impact on the mental health of individuals. By 2050, obesity is predicted to affect 60% of adult men, 50% of adult women, and 25% of children if present trends continue (1). The World Health Organization (WHO) regards childhood obesity as one of the most serious global health challenges for the 21st century (2,3) with a significant burden to society. Obese children have increased metabolic and cardiovascular risks both in childhood and adulthood (4), and may show early signs of the metabolic syndrome, such as dyslipidaemia, hypertension, and disorders of glucose metabolism (5,6). Current treatment approaches are sub-optimal both in adults and children (4). In the last decade, important studies have been conducted aiming to identify strategies to prevent obesity during critical periods of life. The underlying aetiology of obesity is complex, with several risk factors and mechanisms that are interconnected. The weight excess can manifest across the life cycle: mothers who are overweight or obese at the time of conception can transmit effects to their offspring creating intergenerational cycles of obesity (7). The topic of genetic and environmental influences on obesity, and how they interact, is a unique topic for which conceptual frameworks are scarce. Also, the different economical settings have to be considered on the major risk of pediatric obesity (8,9).

The focus of this article is to update and expand on the previous European Society for Paediatric Gastroenterology, Hepatology and Nutrition Committee on Nutrition (ESPGHAN CoN) position paper published in 2011 (10) that evaluated the role of dietary factors and food habits in the prevention of obesity in children and adolescents. Factors in infants and children <2 years of life, as well as the role of physical activity and sedentary behaviour will also be considered in this updated version. The maternal factors and exposures during pregnancy and the dietary management of obesity is beyond the scope of this article.

## METHODS

**FIGURE 1:**
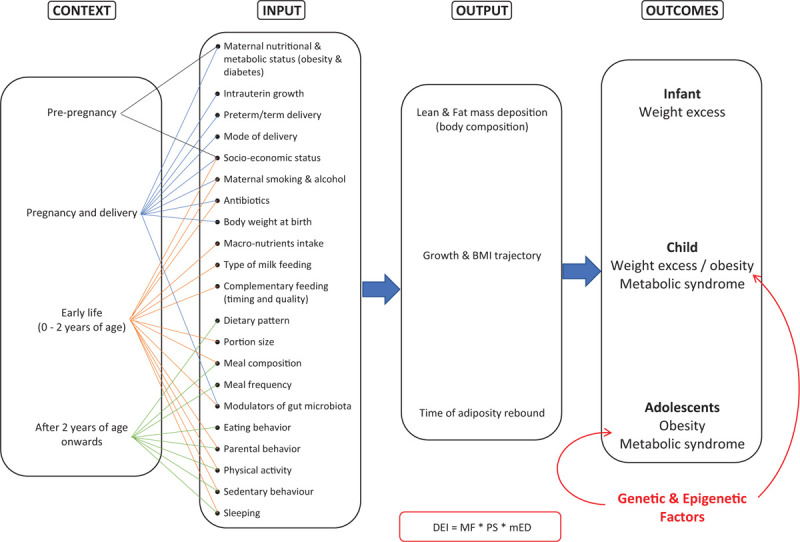
Logic model proposed to examine the complex systems of the child and adolescent obesity development. DEI = Daily Energy Intake; mED = meal's Energy Density; MF = Meal Frequency; PS = Portion Size.

In the present article, we have developed a logic model to highlight some key aspects of the complex system of child and adolescent obesity development (Fig. 1).

We present results from systematic reviews and meta-analysis, randomised controlled trials (RCTs) and large observational studies, published from 2011 onwards, on the possible role of the following factors in obesity development: breast-feeding; macronutrient composition and method of complementary feeding; parental style; dietary patterns; sugar-sweetened beverages (SSBs) consumption; eating behaviour (eg, skipping breakfast, family dinners, etc); meal frequency and composition (fast foods, snacking), portion size; dietary modulators of gut microbiota (including pre-, pro-, and synbiotics); physical activity; and sedentary behaviour (see Table 1).

**TABLE 1:**
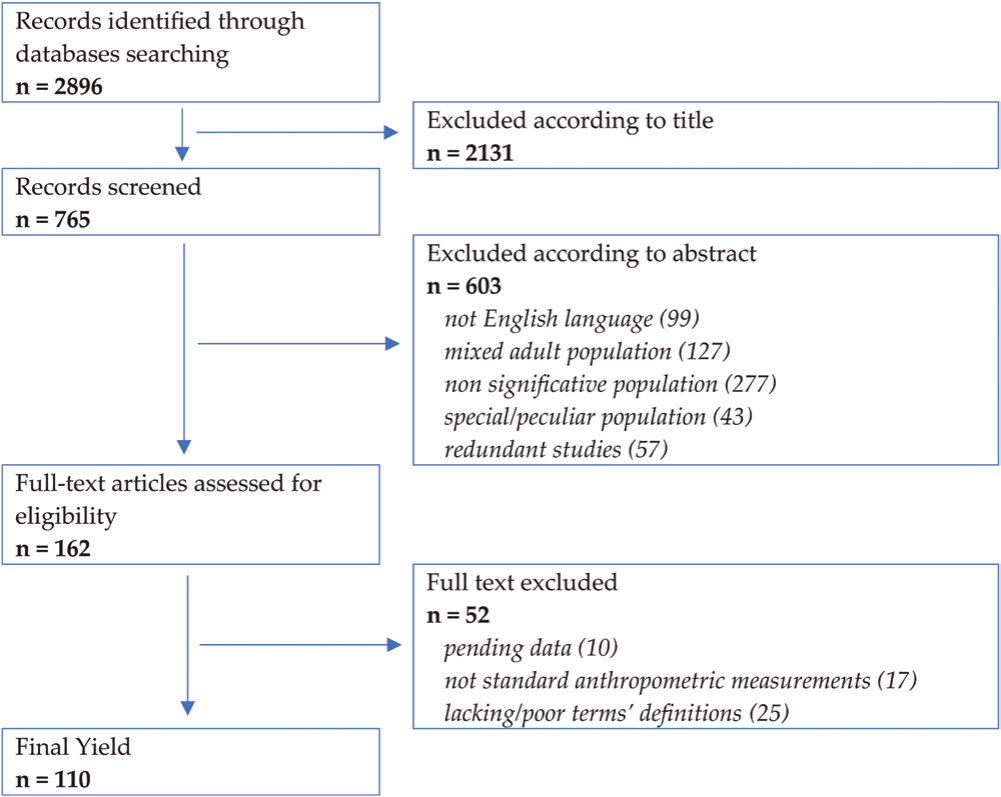
Flowchart with eligibility criteria for inclusion in this Position Paper

In order to retrieve references specifically related to children and relevant to the scope of this position paper, the database Medline (via PubMed) and Cochrane Database of Systematic Reviews were searched for keywords of publications up to March 2020 (Appendix 1, *http://links.lww.com/MPG/C237* ). Only manuscripts published in English were evaluated as full papers.

## FIRST 2 YEARS OF LIFE

### Breast-feeding

Breastfed (BF) and formula-fed (FF) infants have different body composition, measured using a variety of methods (11), with FF infants showing significantly lower fat mass (FM) compared with BF infants in the first 6 months of life (−0.09 kg [−0.18; −0.01 kg] at 3 to 4 months and −0.18 kg [−0.01; −0.34 kg] at 6 months), although the trend toward higher FM in FF infants, observed in the second 6 months of life, was not significant at 12 months. A similar effect for FM% was also documented (11). It is important to note that studies included in this meta-analysis showed marked heterogeneity with respect to study design, techniques used to measure body composition, and measurement timepoints.

Two meta-analyses (12,13) and a review of systematic reviews (14) published data on the association of breast-feeding and childhood obesity prevention. The most recent meta-analysis by Horta et al (12), including studies from high-income and low-/middle-income countries, showed a preventive effect of BF on later overweight/obesity with tight 95% confidence interval (CI) (pooled odds ratio [OR]: 0.74; 95% CI: 0.70--0.78). The meta-analysis by Yan et al (13) also reported a possible preventive effect of BF. In the meta-analysis by Yan et al (13), however, the majority (22/25) of studies were from high-income countries, the definition of obesity was very heterogeneous, and moreover some studies also included individuals who were overweight but not obese.

The review by Stanley did not show a clear effect, perhaps indicating a risk of residual confounding (14).

A more recent systematic review evaluated the association of exclusive and partial breast-feeding duration with growth during infancy (15) and demonstrated that shorter duration of breast-feeding (4 vs 6 months), especially if exclusive, tended to be associated with faster weight gain in high-income country settings, during the first year of life (16,17). It is important to note that an individual-level meta-analysis found that each +1 unit increase in weight standard deviation (SD) scores between 0 and 1 year conferred a 2-fold higher risk of childhood obesity and a 23% higher risk of adult obesity, after adjustment for sex, age, and birth weight (17).

The systematic review and meta-analysis by Giugliani et al (18) included BF promotion intervention studies. Of 35 studies, 16 provided sufficient growth data and were included in the meta-analysis. A modest but significant decrease in mean body mass index (BMI) (or weight for length/height) *z* score (mean difference −0.06 [95% CI: −0.12 to 0.00]) in low- and high-income countries was observed, although this effect was not seen in low-medium income countries. Therefore, intervention studies were inconclusive, and more studies are needed.

Moreover, it should be noted that breast-feeding rates in the United States differ significantly depending upon the income of the mother (19), suggesting that economically poor settings could be early negative marker of the pediatric obesity risk, combining with low breast-feeding rates.

### Complementary Feeding

#### Macronutrient Intake

According to the ESPGHAN Position Paper on complementary feeding (CF) (20), complementary foods (solids and liquids other than breast milk or infant formula) should not be introduced before 4 months but should not be delayed beyond 6 months. In 2019, the EFSA Panel on Nutrition, Novel Foods and Food Allergens concluded that there was no effect of introduction of CFs at 3 to 4 months of age, compared with 6 months of age, on body weight, body length, head circumference, BMI, and body composition (21). It should be noted, however that the limit of 3 months would have a negative impact on breast-feeding in Europe.

In the ESPGHAN Position Paper, the CoN concluded that a high protein intake during CF may increase the risk of later overweight and obesity and recommended limiting the protein intake to 15% of total energy for infants and toddlers (20). After this publication, Appleton et al (22) published a systematic review examining the association between formula feeding practice and excess or rapid weight gain. Where infants are not receiving breastmilk, using a formula with a lower protein content (1.25 g/100 mL standard formula, then 1.6 g/100 mL follow on) from birth might reduce the risk of rapid or excess weight gain and the childhood risk of being overweight or obese (23,24). The authors, however, highlight that the review only included 2 RCTs (23,24,25) (total n = 1262 formula-fed infants), with the Inostroza study (25) including infants born from overweight or obese mothers and using a low-protein formula (1.04 g/100 mL) also containing probiotics. On the other hand, probably the diet's protein content is not only the variant to be considered (26) but also the possible role of the early fat/protein balance should be taken into account as the role of excess energy intakes in case of formula-fed infants, irrespective of the macronutrient balance (27).

A recent systematic review and meta-analysis of cross-sectional and prospective cohort studies evaluated the relationship between regular cow-milk fat consumption (defined as daily or ≥4 times per week) and adiposity in healthy children ages 1 to 18 years (28). Among children who tended to consume whole-fat (3.25% fat) compared with reduced-fat (0.1–2%) milk, the adjusted OR of overweight or obesity was 0.61 (95% CI: 0.52--0.72; *P* < 0.0001). It, however, seems likely that some of the association might be because of confounders or because of reverse causation, for example, because of parents of overweight children choosing lower fat milks.

Limited evidence is present on the effect of young child formula on health outcomes in toddlers (29).

#### Method of Feeding

A modified version of baby-led weaning called Baby Led Introduction to SolidS (BLISS study) was developed to avoid energy and iron deficiency, and the choking hazard potentially associated with baby-led weaning, and was compared with the traditional spoon-feeding approach in a RCT (30). The mean BMI *z* score was not significantly different at 24 months between the 2 groups, although a lower satiety responsiveness and less parent food fussiness was reported in BLISS infants. Also, a more recent RCT (31) failed to show any influence of method of feeding on overweight and obesity at 2 years of age.

### Parenting Style

A recent single-centre RCT evaluated the effect of a responsive parenting intervention among 279 mother-child dyads on BMI *z* score at 3 years (32). Full-term singleton newborns delivered to primiparous mothers ≥20 years and with a birthweight >2500 g were recruited. The intervention consisted of parental advice on their child's behavioural states, focusing on feeding, sleep, interactive play, and emotional regulation. The control group received an intervention on safety that was dose-matched to ensure equivalent time and intensity. The children in the responsive parenting group showed a lower mean BMI *z* score at 3 years compared with controls (absolute difference, −0.28 [95% CI: −0.53 to −0.01]; *P* = 0.04). Further studies with sufficient sample size are needed in order to determine whether this method is effective for the prevention of obesity. The responsive parenting intervention used in this study may affect multiple domains of child behaviour and could be associated with better development of child self-regulation. It should be noted that the present study has been carried out in white, middle-income, predominantly well-educated mothers, and attrition could be more likely to occur among higher risk participants.

## FROM 2 YEARS OF LIFE ONWARDS

### Dietary Patterns

#### Mediterranean Diet

**FIGURE 2:**
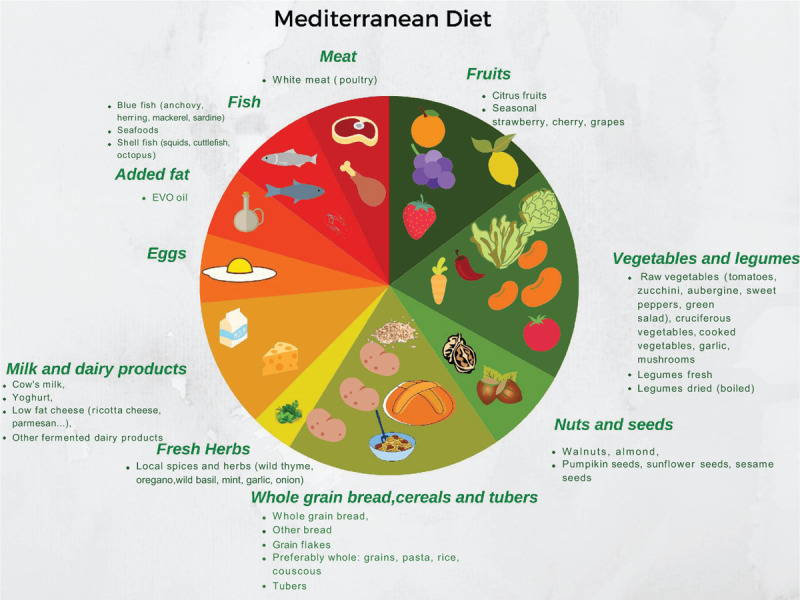
Visual representation of the Mediterranean diet composition.

The Mediterranean diet (MD) is a dietary pattern rich in plant-based foods (vegetables, fruits, whole grain cereals, legumes, nuts, seeds), moderate-to-high intake of fish and seafood, moderate consumption of eggs, poultry, and dairy products (milk, yoghurt, and cheese), and low consumption of red meat. Olive oil, rich in unsaturated n-9 fatty acids, is the main source of added fat (Fig. 2). MD adherence varies widely within the Mediterranean countries for both children and adolescents, and it can be evaluated through quantifying scores or indexes, such as KIDMED, MDS, fMDS, or MediLIFE—questionnaires that vary in the number of category classification systems, components, questions’ content, scoring system, and contribution (positive or negative) of a single component to the total score—that can be self-administered or conducted by interview led by a paediatrician, dietitian, and so forth.

Two systematic reviews (33,34) aimed to summarise the available literature on the dietary intake of European children and adolescents and investigate the association between a Mediterranean-like diet, MD adherence, and nutritional status. Pereira-da-Silva et al (33) focused more specifically on preschool children in the Mediterranean countries of the European Union. A lower adherence to a Mediterranean-like dietary pattern, evaluated by KIDMED index, was associated with a higher prevalence of being overweight and obese, and greater maternal education and familial socioeconomic status was associated with better quality diets. Iaccarino Idelson et al (34) found that the association between MD adherence (assessed with 3 different indices: KIDMED, MDS, fMDS) and weight status was not consistent in 2- to 20-year-old children and adults even if a better diet quality was observed. Nevertheless, a positive association was found with physical activity and a negative association with sedentary behaviour.

More recently, a Greek cross-sectional study (35) involving ∼170,000 children and adolescents (6–18 years) published data on the association between MediLIFE Index scores and anthropometric measurements. This study considered 4 components (the KIDMED index, physical activity level, sedentary time, and sleep duration), and anthropometric measurements. Higher MediLIFE scores were associated with lower BMI, waist circumference and waist-to-height ratio (WtHR), and lower prevalence of overweight, obesity, and abdominal obesity, by 6% (OR 0.94; 95% CI: 0.92–0.98), 30% (OR 0.70; 95% CI: 0.67–0.75), and 20% (OR 0.80; 95% CI: 0.77–0.83), respectively. It is not, however, possible to draw any conclusion from the present study, regarding the role of MD adherence on the risk of overweight and obesity as an index based on different factors was used.

#### Nordic Diet

**FIGURE 3:**
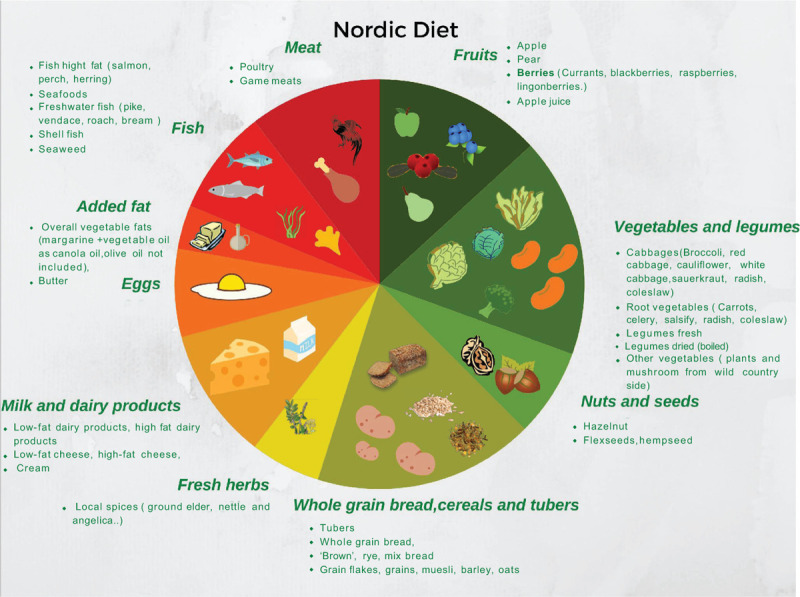
Visual representation of the Nordic diet composition.

The Nordic diet (ND) is a dietary pattern, which refers to a modern dietary profile commonly available in the Nordic regions and acknowledged worldwide only in the last few years. ND is high in fruits and vegetables (especially berries, cabbages, root vegetables, and legumes), plants and mushrooms collected in the wild, fresh herbs, potatoes, nuts, whole grains (mainly barley, rye, and oats), rapeseed oil, fatty fish (especially salmon, herring, and mackerel) and shellfish, seaweed, low-fat and white meat, game, and also emphasises the consumption of low-fat dairy products and avoidance of sugar-sweetened products (Fig. 3). Additionally, similar to MD, the ND mainly focuses on local, organic, and wild food items. Indeed, the main difference of ND compared with other dietary patterns is related to the recommended type of oil, vegetables, and fruit.

There are very few attempts to explore associations between ND adherence and obesity in the pediatric population. Besharat Pour et al (36) estimated the association between parental migration background, nutrition, physical activity, and weight in 8-year-old children born in Stockholm, including offspring of immigrants. Using a Food Frequency Questionnaire, ND adherence was assessed based on the compliance of nutrients intake as stated in the “Nordic Nutrition Recommendations of 2004” guidelines (37). Offspring of immigrants complied more fully with nutritional recommendations but had a higher risk of having low physical activity and hence being overweight compared with children of Swedish origin (36).

An intervention study (38) investigated the effects of introducing hot school-meals, following the principles of the ND, instead of packed lunch (traditionally based on cold sandwiches) in a cohort of Danish children ages 8 to 11 years. ND adherence was established using a self-administered internet-based interactive food record tool. No difference was found in the average daily energy intake but beverage intake was lower. Moreover, a lower energy intake from fat and a higher energy intake from protein, vitamin D and iodine was observed (38), although the effect on obesity was not evaluated.

#### Vegetarian Diet

**FIGURE 4:**
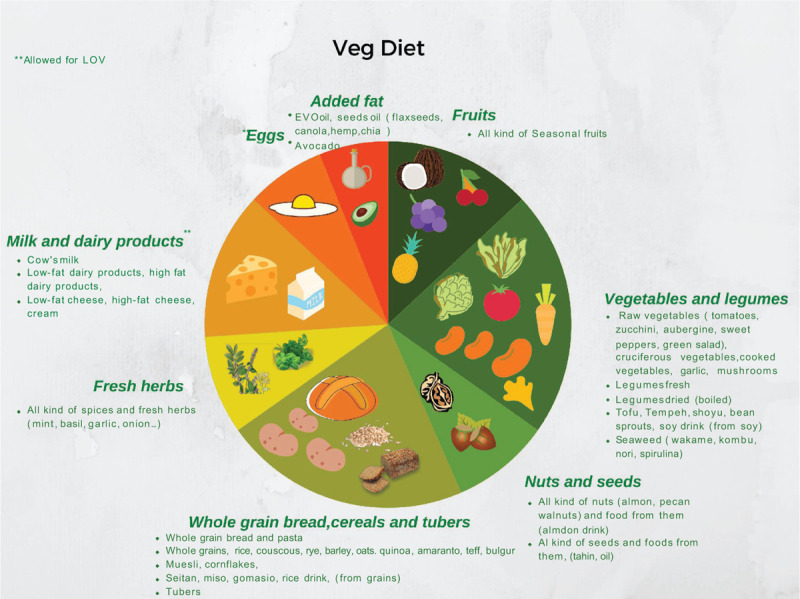
Visual representation of the vegetarian diet composition.

Two main types of vegetarian diet are recognized (39) (Fig. 4).

##### Lacto-ovo-vegetarianism

Lacto-ovo-vegetarianism (LOV) excludes meat but includes dairy products, eggs, and honey, together with a wide variety of plant foods. Subcategories are lacto-vegetarianism (LV), which excludes eggs, and ovo-vegetarianism (OV), which excludes dairy products.

##### Veganism

Veganism (VEG) excludes meat, dairy products, eggs, and honey.

Vegetarians were estimated in 2018 to represent about 8% of the world population (40). Table 2 reports the prevalence of LOV and VEG in certain countries (2016–2019 data). The exact number of vegetarian children is not known but it is likely that many vegetarian parents would raise children following a similar dietary pattern.

**TABLE 2:** Prevalence of lacto-ovo-vegetarianism and veganism in some worldwide countries (2016--2019 data)

Country	LOV, %	VEG, %
India^∗^	31	
USA^†^	5–8	3
Germany^‡^	10	1.6
Italy^§^	5.4	1.9
Spain^¶^	1.3	0.2
UK^||^	7	1.16
France^#^	5	0.25
Greece^∗∗^	2	0.8
Switzerland^††^	14	3

LOV = lacto-ovo-vegetarianism; VEG = veganism.

∗ *http://censusindia.gov.in/vital_statistics/BASELINE%20TABLES07062016.pdf* .

† *https://news.gallup.com/poll/238328/snapshot-few-americans-vegetarian-vegan.aspx* .

‡ *https://proveg.com/de/confirmation/veggie-fakten-nicht-mehr-verfuegbar/* .

§ *https://eurispes.eu/rapporto-italia-2019-vegetariani-e-vegani-le-nuove-diete-si-consolidano/* .

¶ *https://web.archive.org/web/20170625180431/http://www.lantern.es/wp-content/uploads/2017/02/infog.jpg* .

|| *https://www.plantbasednews.org/news/veganism-skyrockets-to-7-of-uk-population-says-new-survey* .

# *https://web.archive.org/web/20180703120048/https://o.nouvelobs.com/food/20180703.OBS9086/en-france-le-vegetarien-est-plutot-une-femme-trentenaire.htmL* .

∗∗ *https://www.protothema.gr/ugeia/article/870879/go-vegan-80000-atoma-aspazodai-ti-diatrofi-stin-ellada/* .

†† *https://www.swissveg.ch/veggie_survey?language=en* .

There is limited literature that explores the effect of vegetarian diets in children on the risk of overweight and obesity. The limited evidence available indicates that the growth of LOV children (41,42) and adolescents (41,43–45) is comparable to that of their omnivoric (OMN) peers. Furthermore, data suggest that VEG children tend to grow in a similar pattern to non-VEG children (41,46).

A more recent study (47) conducted on 215 healthy adolescents, attending 5 Adventist secondary schools in Australia, showed that students consuming predominantly vegetarian foods had a significantly lower BMI. In this study, adolescents were classified as vegetarians if they consumed red meat, chicken, and fish less than once a week. It is important to note that no adjustment for confounders was performed and the classification used for vegetarians and nonvegetarian adolescents does not enable an exploration of the effects of vegetarian status following either a LOV or a VEG diet.

Inadequate intake of energy, protein, calcium, zinc, iron, vitamin B12, and vitamin D may occur on a vegetarian diet because of a limited variety and sub-optimal choice of foods (39,48). Therefore, whenever a vegetarian diet is used for children, appropriate nutritional planning and monitoring is recommended to be supervised by an adequately trained health care professional.

#### Sugar-sweetened Beverages

SSBs are beverages or drinks that contain added caloric sweeteners (ie, sucrose, high-fructose corn syrup, fruit juice concentrates). The ESPGHAN CoN recently published a position paper (49) that evaluated outcomes related to the intake of sugar in infants, children, and adolescents, and provide recommendations and concluded that a higher than recommended intake of free sugars (ie, mono and disaccharides), particularly SSBs in children and adolescents, is associated with an increased risk of excess weight gain.

A recent systematic review (50) of prospective cohort studies and RCTs found a positive association between consumption of SSBs and weight in children. In particular, 16/17 prospective cohort studies, none of which were funded by industry, showed a positive association of SSBs on obesity, and 3 RCTs demonstrated that SSB consumption had an effect on BMI or BMI *z* score. The RCTs explored the effect of 2 nutritional education programs at school (1 giving general health and advice on healthy eating and 1 focused on reducing SSBs consumption by encouraging water), and 1 healthy lifestyle education program. Two out of 3 of these trials adjusted for physical activity but no study adjusted for dietary energy intake.

A systematic review of 27 intervention studies aiming to reduce SSBs consumption in children ages 0 to 5 years showed that the interventions conducted in preschool/daycare settings, specifically targeting only SSBs or only oral hygiene, were able to reduce the SSB consumption (51). The variation in study characteristics, design, and reporting of results, however, make it difficult to compare effectiveness of strategies across studies.

Fruit juices (100% fruit part) are not considered SSBs. No evidence was found to support a positive association between fruit juice consumption and weight gain (52,53), and there is currently a lack of RCTs on this topic. Even if fruit juices tend to have a superior nutritional composition compared with SSBs containing minerals and vitamins, the amount of free sugar and energy is, however, similar to those of SSBs, which may have similar effects on weight gain (49).

### Dietary Modulation of Gut Microbiota

Gut microbiota play an important role in the absorption, storage, and utilization of energy obtained from diet. Furthermore, the gut microbiota is also involved in the regulation of food intake by affecting hormones that influence metabolic function and specific brain areas associated with eating behaviour (54). This so-called “gut microbiota-brain axis” represents a bidirectional signalling axis that may contribute to body weight by influencing appetite, storage, and energy expenditure (55,56).

One of the key activities of the gut microbiota is through the harvesting of energy for the host through fermentation of otherwise indigestible nutrients, including oligo- and polysaccharides, and production of short-chain fatty acids (SCFAs), that is, acetate, propionate, and butyrate. Among their multiple actions, butyrate acts as an energy substrate for colonocytes, acetate contributes to de-novo lipogenesis, and propionate is metabolized in the liver, regulating production of appetite hormones and cholesterol metabolism (57).

The causal relation between gut microbiota composition and energy homeostasis is, however, complex and is largely based on preclinical research and/or association studies. Multiple other factors are important in the aetiology of obesity, including genetic, epigenetic, and gene-nutrient interactions, which may also be important modifiers of gut microbiota structure and function (58,59). Also, the maternal pre-pregnancy BMI may have an impact on infants’ gut microbiome (60).

Childhood obesity, as in adults, has been associated with an increased Firmicutes/Bacteroidetes ratio in faeces and reduced microbial diversity and richness in the gastrointestinal tract compared with normal-weight, although it is difficult to determine the effect of residual confounding or reverse causation (61,62).

Recently a 4-year prospective study of 70 school-age children, evaluated the association between clusters of dietary habits and gut microbiota diversity and whether the interaction of microbiota-host-diet may predict obesity (63). Out of 70 normal-weight children at start, 34 remained normal-weight and 36 became obese. The combination of “high carbohydrate/high fat” or “high protein/high fat” dietary pattern and low diversity of microbiome was associated with the onset of obesity. Furthermore, the study suggests that the individual gut microbiome configuration and long-term dietary habits together can be considered as a predictive tool for the development of obesity in children (63).

Considering that lifestyle changes are difficult to implement over the longer term, research efforts have also explored alternative strategies, such as probiotics and/or prebiotics in order to modulate gut microbiota and prevent obesity.

Luoto et al (64) conducted an RCT in 159 women of *Lactobacillus rhamnosus* GG supplementation (1 × 10^10^ CFU) commenced 4 weeks before expected delivery combined with treatment of the child during the first 6 months of life and showed slower weight gain in children at 1 and 4 years of age in a secondary post hoc analysis, although no evidence of long-term effects (10 years) was found. It is, however, important to note that the primary aim of the study was to investigate the preventive effect of probiotics on the onset of allergic diseases, and therefore, multiple different confounding factors that may influence the obesity risk may have been present. A more recent RCT (65) randomized 179 term-born, vaginally delivered infants to receive cereals without or with probiotic (*Lactobacillus paracasei* ssp F19 [1 × 10^8^ CFU]) between 4 and 13 months of whom 120/179 were followed until 8 to 9 years. No significant differences were found in BMI *z* score and body composition, measured using dual energy X-ray absorptiometry.

We found no published studies on the effect of prebiotics or synbiotics on prevention of childhood obesity.

### Eating Behaviour

#### Skipping Breakfast

Skipping breakfast, defined as not eating between 06:00 and 09:00 a.m., has always been considered a risk factor for obesity as it is believed to have a critical role in energy balance and dietary regulation.

A recent systematic review (66) of observational studies aimed to summarise the association of skipping breakfast with body weight and metabolic outcomes in children. The total sample included 286,804 participants (2–18 years) living in 33 countries. Data were appeared consistent perhaps as the studies represented children living in multiple different locations; however, definition and assessment of overweight/obesity and of skipping breakfast were highly heterogeneous. Regardless of this, most of the studies reported that at least 10% to 30% of children and adolescents never ate breakfast, and there was an increasing trend in skipping breakfast from childhood to adolescence, as well as reporting higher values in girls than in boys. Overall, studies representing around 94% of all subjects reported a positive association between skipping breakfast and obesity. In conclusion, this review supports that skipping breakfast may be a useful predictor of the risk of overweight/obesity, even if the mechanism of weight excess may be because of a higher energy intake during the following hours in children who skipped breakfast (66).

Traub et al (67) collected data about skipping breakfast within a population of German primary schoolchildren. Regression model for the prevalence of abdominal obesity, overweight, and obesity at 1 year follow-up showed a significant association between skipping breakfast and abdominal obesity (according to WtHR) and overweight (OR = 3.36, 95% CI: 2.23–5.07; *P* = 0.006 and OR = 2.30, 95% CI: 1.54–3.45; *P* = 0.034, respectively) (67). Girls skipped breakfast significantly more often than boys (15.2% vs 10.6%).

An Italian study (68) included a representative sample of 11- to 15-year-old children from 20 Italian regions who completed a self-reported anonymous questionnaire indicating, in a typical week, how many days they had breakfast (defined as having more than a glass of milk or fruit juice). The 2 categories “daily breakfast consumption” (7 days in a week) and “less than daily” (less than 7 days in a week) were considered. Authors found that girls more often skipped breakfast than boys (“less than daily”: 55.9% vs 48.6%). Among all age groups and both in girls and boys, “less than daily” breakfast consumption was associated with overweight (OR = 1.33, 95% CI: 1.16–1.51 in boys; OR = 1.58, 95% CI: 1.38–1.82 in girls). Summarizing, no daily breakfast consumption was associated with overweight including obesity (68).

The positive association between skipping breakfast and weight status, reported in these studies, may nevertheless reflect “reverse causality,” considering that children with greater body mass may eat less frequently.

#### Family Dinner

In a systematic review conducted by Valdés et al (69), frequent family meals (and therefore, dinner) have been associated with a healthier and more varied dietary pattern. Six out of 11 cross-sectional studies and 1 out of 4 longitudinal studies found statistically significant inverse associations between frequent family dinner and being overweight. Most of the cross-sectional studies showed this inverse association was more consistent among children than adolescents. These results also showed how the potential protective effect of family meals may be limited to younger children (4–7 years old).

Valdés et al (69), however, noted that 1 of the 3 longitudinal studies found an inverse association between frequent family dinner and overweight that approached significance among middle-school girls. This potential association could reflect the greater incidence amongst girls, compared with boys, of having an eating disorder, such as binge eating and dieting. Irrespective of the findings, however, all studies suffered from 2 major limitations: the lack of a standard definition and characteristics of family meal.

More recently, a sub-study of a project done by Roos et al (70) among 11-year-old European children, found that having family meals (mainly breakfast and dinner) and TV viewing during dinner was not associated with overweight. When these associations were, however, stratified by region, results showed that in Northern Europe, children who had family breakfast or dinner less than once weekly and TV viewing during the dinner were more likely to be overweight, while there was no association between family breakfast or dinner and adiposity status (according to BMI) in Southern and Eastern European countries (70). These country discrepancies may be explained by different dietary patterns and lifestyles across Europe.

In 890 young Japanese adolescents, Shirasawa et al (71) observed no difference in terms of eating dinner alone between overweight and nonoverweight boys. Compared with girls not eating dinner alone, girls who ate dinner alone ≥1 time/week showed an increased risk of overweight (adjusted OR = 2.78; 95% CI: 1.21–6.38).

Haghighatdoost et al (72) studied a sample of 5528 Iranian adolescents and showed that no significant differences were found in dietary intake between family dinner “consumers” (≥5 times/week) and “skippers” (<5 times/week). After controlling for confounders, family dinner consumers were, however, associated with reduced odds for central obesity (according to WtHR) by more than 30% and reduced odds for obesity (OR = 0.67, 95% CI: 0.5--0.96).

### Meal Frequency and Composition, Portion Size

#### Eating Frequency

Two large observational studies and a meta-analysis of 11 cross-sectional studies showed that a higher number of daily meals is associated with a lower risk of obesity in children.

Zurriaga et al (73) conducted a matched case-control study on 1188 Spanish children, ages 2–14 years, and observed that consuming 5 meals per day was associated with lower childhood obesity risk (73).

In a cross-sectional, multi-centric survey within 13,486 Iranian children and adolescents, ages 6 to 18 years, Kelishadi et al (74) noted that as age increased, eating frequency (EF) decreased: 13-year-old students ate ≤3 meals and/or snack, whereas 11 years old participants ate ≥6 meals and/or snacks during the day. Anthropometric indices, such as weight, waist circumference, and BMI, were higher among those who had an EF of ≤3 compared with those with EF ≥6. An obese status was observed in 14% of students who reported EF ≤3, and in 9.5% of those with EF ≥6. Concerning the risk of central obesity, a significant inverse association with EF has been shown; having an EF of 4, 5, or ≥6 decreased the abdominal adiposity risk, even though, higher EF might be expected to lead to excess weight through higher daily caloric intake. The authors of this study suggested a role in the reduction of hunger provided by higher and regular EF (74).

A large meta-analysis of 11 cross-sectionals studies involving 18,849 children, 2 to 19 years of age, showed a modest but negative association of daily EF with weight status (75). In 5 out of the 11 studies, the EF associated with lowest weight was 5 meals per day. It is important to note that many of the studies in this meta-analysis suffered from limitations inherent to dietary assessment methods.

Snacks are defined as a small portion of food given or consumed in-between main meals, frequently with an intention of reducing or preventing hunger until the next mealtime (76). The American Academy of Pediatrics (AAP) recommends 2 snacks daily for preschool-aged children as part of obesity prevention (77).

Snacking frequency (more than recommended by AAP) has been positively associated with weight among preschool children in the 2005 to 2014 National Health and Nutrition Examination Survey (NHANES), taking into account the dietary reporting bias (78). Normal-weight children tended to snack less frequently than children with adiposity excess when considering all foods/beverages eaten between meals (78). The observed mean effects were very small (3.2 vs 3.3) but these findings raise the possibility that small differences in snacking may accumulate over time to influence obesity risk among young children. Similar results were observed in a recent NHANES analysis of snacking and weight status among older children (6--11 years old) (79).

#### Meal Composition: Consumption of Fast Foods, Snacks

Fast-food consumption is increasingly considered a contributing factor for increasing obesity prevalence in childhood. Despite this, a recent systematic review and meta-analysis of longitudinal and cross-sectional studies (80), focusing on fast-food restaurant (FFR) access and childhood obesity, reported a lack of association in most studies when BMI-related continuous measures were used. Whenever using overweight/obesity outcomes, about half of the cohort studies and one-third of the cross-sectional studies reported a positive association but no significant results were observed in separate meta-analyses between various measures of FFR access and body weight. The authors conclude that this systematic review was limited by methodological diversity of the different studies (80). A study performing comparative analyses in 2 German pediatric cohorts (total n = 670 children), the “Kiel Obesity Prevention Study” (KOPS) and the “Identification and prevention of Dietary- and lifestyle-induced health EFfects In Children and infantS” (IDEFICS-Germany) studies, however, confirmed an adverse impact of fast-food consumption on excess weight gain during primary school years (81). Furthermore, a secondary analysis from a multi-centre, international cross-sectional study (International Study of Asthma and Allergies in Children [ISAAC] Phase Three) found an association between increasing frequency of fast-food consumption and higher BMI in 6- to 7-year-old children but in female adolescents, a higher frequency was associated with a lower BMI (82). This latter result could be because of bias, particularly underreporting of fast-food consumption and reverse causality in adolescents.

It is important to point out that multiple confounders in the food environment, such as large supermarkets, convenience stores, fast-food outlets, and children's age may influence this association.

Recently, a large cross-sectional study (83), using data from the Yorkshire Health Study (n = 22,889), suggested that the association between fast-food outlets and obesity varies by age. Indeed, with increasing age, the highest availability of fast-food outlets has been associated with risk of obesity.

A previous systematic review investigated the associations between food outlets near schools and children's food consumption but no firm conclusions were made (84). A subsequent study conducted in Arkansas, USA (one of the poorest and least healthy US states), suggested that the number of FFR within 1 mile can increase school-level obesity rate (85). This might also apply in European settings, where school days typically end mid or late afternoon when hungry children leave school and either go to a nearby FFR or consume fast-food on the way home. Well planned studies are, however, needed to test this hypothesis.

The association of snacking with overweight/obesity is unclear, particularly for young children for whom snacks are believed to be the most nutritionally important (86). The problem is not only the frequency but also portion size and the type of foods consumed during snacking. Indeed, snacking contributes to increased energy intake in children, and the energy density (ED) of snacks has increased in recent decades (87,88). Snack foods eaten by children increasingly consist of foods, such as desserts, sweetened beverages, and salty snacks that also tend to be high in ED, saturated fat, and refined sugars.

A RCT (89) reported the efficacy of a school-based intervention, aiming to improve the nutritional value of snacks on the dietary intake and waist circumference in 1433 Ecuadorian adolescents. A decreased consumption of table sugar, sweets, salty snacks, fast foods, soft drinks, and packaged foods parallel to a reduction of waist circumference were observed after 28 months in the intervention group.

Recently, a systematic review of cross-sectional, longitudinal, and experimental studies (90), observed that parental restrictive feeding and home access to foods with high ED, saturated fats, and added sugars were consistently associated with snacking among children ages 2 to 18 years.

#### Portion Size

Three different systematic reviews (91–93) evaluated portion size as a determinant of obesity risk. In Rolls’ systematic review (91) several experimental studies testing the responsiveness to increasing portion size were reported. Three years old children appeared largely unaffected, whereas 5 years old children consume more as portion size increases but additional studies have failed to clearly demonstrate such developmental changes in the susceptibility to portion size. Of note, 1 study showed that children who were allowed to serve food themselves, ate 25% less of a large main course compared with those who were served the large portion by an adult. In addition, 4 years old children taught to focus on self-regulatory satiety cues (such as the fullness of their stomachs) showed better self-regulation of energy intake than those who were rewarded for completing their plates. Although there is convincing evidence that portion size has persistent effects on energy intake, data do not prove that portion size alone plays a role in the aetiology of obesity.

In a further systematic review, Small et al (92) focused on 2 main themes: to determine the effect of varying portion size and ED of food on energy or food intake and to determine the child's age at which variable portion sizes can affect the dietary intake. Even this review, however, pointed out that overall energy intake was affected by the size of the served portion, with larger served portions resulting in greater daily energy intake in many of the children. In addition, ED and portion size positively affected the daily energy intake, as the serving and consumption of energy-dense foods resulted in increased energy intake. An interesting finding was that larger portion sizes of vegetables resulted in greater vegetable intake, even if not affecting the amount of the other foods subsequently consumed. The age of the studied populations, included in this systematic review, ranged from 2 to 9 years. The age at which young children could override internal self-limiting mechanisms and might become sensitive to larger portions was not well described as study findings were equivocal for children who were 2 and 3 years of age. Children 4 years and older, however, demonstrated increased energy intake when large portions were served. Another important finding is that a parent-directed intervention regarding the children portion education appears to be successful only if parents are able to learn how to estimate portion sizes. Interestingly, school-aged children (8–12 years) who directly underwent the same intervention did not learn this skill.

A review by Birch et al (93) suggested that, although there was a positive correlation between portion size and weight status, data did not support the idea that large portions were causally implicated in the development of greater BMI and obesity onset, and the positive relation between portion size and weight status could reflect “reverse causality.” Children with greater body mass consumed large portions as their energy requirements were greater. Although children's preferences (likes and dislikes) are primary determinants of what and how much food is consumed, the effects of preferences and palatability have not been systematically studied. Although additional research is needed, findings suggested that “liking” or palatability may play a role in determining whether increasing portion size increases children's food intake.

Moreover, a variety of factors, including media, marketing, observational learning of parents and others’ eating behaviour, parents’ feeding practices, and postprandial feelings following the consumption of various portion sizes, are likely to be involved in determining how children learn about portion size.

### Physical Activity and Sedentary Behaviour

A negative association between levels of physical activity and overweight/obesity in pre-school, school-age children, and in adolescents has been shown in prospective studies (94,95).

According to WHO recommendations (96,97), based on observational and intervention trials, moderate-vigorous physical activity (MVPA) should be encouraged for at least 60 minutes daily in all subjects of 3–17 years of age to maintain healthy status.

In pre-school aged children, a RCT (98) conducted in Swedish child health centres in the context of routine health care, aimed to reduce the prevalence of obesity as the primary outcome. The intervention program was performed in 1355 children, starting from 8 to 9 months of age and ending at age 4 years, and was based on the promotion of healthy food and physical activity habits using motivational interviewing and principles from cognitive behavioral therapy. After 1 year of follow-up, there were no differences in BMI between the 2 arms of intervention.

In a European multi-centre study cohort, a secondary analysis of 419 11-year-old children revealed that meeting WHO physical activity guidelines appeared adequate to prevent excess weight in children. Moreover, the authors suggested that recommending 15 to 20 minutes of vigorous physical activity every day could further help to reduce childhood overweight status (99). In the same cohort, data showed that over the course of 5 years (6–11 years), children who spent a longer time in sedentary behaviour had a higher BMI, even when adjusting for time spent in MVPA. This observation supports the concept that inactivity is an independent risk factor for childhood obesity (100). In the same cohort, a longer daily screen time from the ages of 3 to 6 years is associated with a higher BMI *z* score (*P* = 0.002) and WtHR (*P* = 0.001) at 6 years of age. Specifically, for every additional hour per week of inactivity, the risk for overweight and obesity increased by 7% and 10%, respectively (101).

A meta-analysis of observational studies published in 2014 (102) concluded that the degree of physical activity and sedentary behaviour are independent risk factors for obesity, although there is an inverse but weak correlation between the 2 behaviour patterns (*r* = −0.108, 95% CI: −0.128 to −0.087).

A cross-sectional study by the WHO European Childhood Obesity Surveillance Initiative (103), including primary-school children (6–9 years) in 5 European countries, evaluated a “food-risk behaviour score” and a “physical activity-risk behaviour score,” created for each child based on the presence of 8 food-related and 5 physical activity-related (including screen time and sleep duration) health-risk behaviours, respectively. Surprisingly, only 4 out of 13 health-risk behaviours were directly associated with obesity and 3 were even found to be negatively associated with obesity or overweight. In contrast “physical activity-risk behaviour score” correlated directly with obesity, confirming that both physical activity and sedentary behaviour have a key role in the energy expenditure balance.

Data show that adolescents tend to have sedentary behaviour and low physical activity more frequently than children, as indicated by a cohort study of 2312 people (104). With regard to sedentary behaviour, a meta-analysis of prospective studies (105) showed a linear dose-response relationship between television (TV) watching and childhood obesity, with an increased risk of 13% for each 1 hour/day increment in TV time. A more recent long-term study (106) showed that TV viewing of at least 2 hours/day versus no TV at the age of 3 to 5 years was associated with an increased risk of overweight and obesity at 5 and 10 years (for 2, 3, and ≥4 hours of TV viewing per day, adjusted ORs were 1.16 [95% CI: 1.00--1.35], 1.39 [95% CI: 1.15--1.69], and 1.61 [95% CI: 1.20--2.17], respectively). Furthermore, a behavioural pattern of high TV viewing time/low physical activity level versus low TV viewing time/high physical activity level at ages 3 to 5 years has been with a risk of overweight/obesity at 5 and 10 years (106). Moreover, eating while TV viewing has been positively associated with childhood and adolescence overweight (OR = 1.28; 95% CI: 1.17--1.39) in a recent systematic review and meta-analysis of observational studies (107). Subgroup analyses showed similar positive associations in children who ate dinner while watching TV (107).

Two meta-analysis of randomised intervention trials (108,109) and a recent review of systematic reviews (110) of studies aimed at reducing sedentary behaviour in children and adolescents, showed a pooled significant but small, reduction of BMI and BMI *z* score. It is important to note that in the Azevedo meta-analysis (109) and in the Reilly review (110) interventions targeting exclusively sedentary behaviour, or sedentary behaviour combined with physical activity, or sedentary behaviour with other behaviours (eg diet, sleep) were included. Research about physical activity in the prevention of obesity has key gaps. Many studies differ considerably in methodology meaning direct comparison is difficult, and even well-designed studies will fail to evaluate the impact of every possible aetiological factor in the obesogenic environment. Thus, many studies were conducted on cohorts including normal weight, overweight, or obese children.

## CONCLUSIONS

### General

Considering the complexity of the development of obesity in children and adolescents, an integrated multi-component approach is required for obesity prevention.

Parental eating behaviours are a key determinant of childhood obesity.

### First 2 Years of Life

- Breast-feeding compared with not breast-feeding has been associated with a preventive effect on later overweight/obesity.
- In high-income country settings, 6 versus 4 months breast-feeding duration is associated with slower growth rates during infancy, especially if exclusive breast-feeding.
- Compared with breast-feeding, formula feeding is associated with altered body composition in infancy.
- Very small (if any) effects of breast-feeding promotion interventions on growth are reported.
- A high protein intake during complementary feeding increases the risk of later overweight or obesity.
- After 1 year, a whole fat cow's milk has been associated with a lower risk of childhood overweight or obesity, compared with reduced fat cow's milk (although this may be because of reverse causation).
- A modified version of baby-led weaning is associated with better food intake self-regulation, without an effect on BMI *z* score at 24 months of life.
- A responsive parenting intervention during infancy is associated with lower BMI *z* scores at 3 years.

### From 2 Years Onwards

#### Dietary Patterns, Sugar-sweetened Beverages Consumption

- Mediterranean and Nordic diets may be a promising approach for obesity prevention, but no firm conclusions can be drawn from the available literature.
- There is insufficient evidence to determine the role of vegetarian diets for the prevention of childhood obesity.
- SSBs consumption is positively associated with the development of obesity.

#### Eating Behaviour

- Skipping breakfast is associated with obesity, possibly because of its role in energy balance and dietary regulation.
- Regular family meals are associated with positive health outcomes and weight excess prevention.

#### Meal Frequency and Composition, Portion Size

- A higher number of daily meals is associated with a lower risk of obesity in children perhaps because of better modulation of hunger.
- During snacking, the consumption of high energy density foods contributes to obesity in childhood, by increasing daily energy intake.
- Larger portion size, especially of energy-dense foods, is associated with greater daily energy intake, a predictor of body weight excess.
- Children can effectively self-regulate energy intake from at least the age of 4 years and up to 12 years, although energy intake tends to increase with increasing portion size.

#### Physical Activity, Sedentary Behaviour

- A combination of increased physical activity and decreased sedentary time may have an important role in obesity prevention.

## RECOMMENDATIONS FOR ROUTINE CLINICAL PRACTICE

Considering the currently available evidence, the Committee of Nutrition of ESPGHAN recommends:

### First 2 Years of Life

- Breast-feeding should be promoted as long as possible during infancy.
- High protein intake must be avoided during complementary feeding.
- There is insufficient evidence to make any firm recommendations about a baby-led weaning approach in terms of childhood obesity prevention.
- Between 1 and 2 years of life, there is no evidence to recommend a reduced-fat cow's milk to prevent childhood overweight or obesity.
- An early response parenting intervention may be included in the multi-component approach for childhood obesity prevention.

### From 2 Years of Life Onwards

#### Dietary Patterns, Sugar-sweetened Beverages Consumption

- Dietary patterns based on the principles of the Mediterranean diet, can be used as the best approach for obesity prevention in the paediatric age group. This recommendation is based on expert opinion as evidence is lacking.
- Public health policies, including correct information for parents through schools, TV, and other media, and by local health professionals, should aim to reduce the consumption of SSBs in preschool and school children, and in adolescents, while encouraging healthy alternatives, such as water. Removal of vending machines selling SSBs where children have access during school breaks, and providing the children with free drinking water, should be the goal.

#### Dietary Modulators of Gut Microbiota

- There is insufficient evidence to recommend the use of pro-, pre-, or synbiotics for obesity prevention.

#### Eating Behaviour

- Children and adolescents should be encouraged to consume breakfast every day.
- Family meals should be promoted; in particular for adolescents, and family dinners should be consumed at least 5 times per week.

#### Meal Frequency and Composition, Portion Size

- Children up to the age of 12 years are encouraged to eat at least 5 meals per day, including a mid-morning and a mid-afternoon snack. Whether eating 6 or more meals per day provides an additional contribution to the prevention of overweight/obesity remains to be elucidated.
- Healthy food options (fruit and vegetables) should be promoted for snacking while avoiding consuming high energy density foods (chips, cookies, sweets).
- Parents need to be educated on healthy food choices and appropriate food portion sizes and they must share this information with their children.

#### Physical Activity, Sedentary Behaviour

- Children (>3 years) and adolescents should spend on average 60 minutes a day on moderate-to-vigorous physical activity.
- Screen time and sedentary behaviour, should be limited in children and the use of screen devices should be avoided during mealtimes.

## RECOMMENDATIONS FOR FUTURE RESEARCH

Considering the currently available evidence, the Committee of Nutrition of ESPGHAN recommends future research should focus on:

### First 2 Years of Life

- Additional studies to assess the role of excess energy intakes, irrespective of macronutrients balance, on later overweight/obesity in case of formula-fed infants.
- Well-designed intervention trials that evaluate the effect of reduced-fat intake on childhood overweight or obesity prevention.
- Conducting large studies to determine whether responsive parenting interventions are associated with better development of child self-regulation.
- Studies to design individualised interventions for obesity prevention from early life.

### From 2 Years of Life Onwards

#### Dietary Patterns, Sugar-sweetened Beverages Consumption

- Conducting high-quality intervention studies to evaluate the effect of childhood obesity prevention in different populations and according to different sex.
- Development of a unified and universally validated score for MD adherence in terms of reproducibility and consistency.
- The effect of taxation on SSBs on the reduction of obesity development in different countries.
- Studies that determine how best to reduce the amount of free sugars in solid foods.

#### Dietary Modulation of Gut Microbiota

- Identify the different dysbiosis patterns in the first 2 years to develop individualised interventions.
- High-quality RCTs that evaluate the effect of dietary modulators of gut microbiota (pre-, pro-, synbiotics) on obesity risk in children.

#### Eating Behaviour

- Interventional studies that confirm whether skipping breakfast in childhood and adolescence is causally related to adiposity.
- Intervention studies to identify how to modify/change the psychological process involved in eating behaviour (decision-making, impulsive behavior, …).

#### Meal Frequency and Composition, Portion Size

- Determining the key aspects of snacking that may influence the risk of obesity, including the quality, timing, and portion sizes of snacks offered.
- Identifying the key parenting behaviours around childhood snacking, which may be used as targets for promoting good health.
- Longitudinal studies evaluating the food environment especially in school neighbourhoods. Policymakers should consider the impact of any planned fast-food outlet interventions according to the likely presence of children of different ages.
- High-quality studies that evaluate the role of factors, such as age, race, ethnicity, income, food insecurity, liking, palatability, and weight status (of both children and parents) and how these may interact with portion size.
- Evaluation on approach of shifting from restriction to more-positive messages relating to the increase of healthy, low-ED foods intake, on obesity risk.

#### Physical Activity, Sedentary Behaviour

- High-quality studies that evaluate the combined effects of physical activity and sedentary behaviour interventions in normal weight children and impacts on obesity prevalence.
- High-quality studies that evaluate vigorous physical activity to reduce the risk of overweight and obesity to determine the types of physical activity that are most beneficial according to the child's age.

## Supplementary Material
