# Supplementary material for: Role of Dietary Factors, Food Habits, and Lifestyle in Childhood Obesity Development: A Position Paper From the European Society for Paediatric Gastroenterology, Hepatology and Nutrition Committee on Nutrition
Source: J Pediatr Gastroenterol Nutr. 2021 Feb 22;72(5):769–83. doi: 10.1097/MPG.0000000000003075 (PMC9770153; doi:10.1097/MPG.0000000000003075)
Supplement: Supplementary file 1 [file jpga-72-769-s001.docx]

The following search terms were used

1. For the “INTRODUCTION” section of the paper (Medline only):

(“children”[Title] OR “childhood”[Title] OR “infant”[Title] OR “adolescent”[Title]) AND (“obesity”[Title] OR “overweight”[Title] OR “metabolic syndrome”[Title])

- with limits to “Full text”, “Books and Documents”, “Review”, “Systematic Reviews” and from 2011 to 2020, March

This search revealed 949 results, out of these 10 were relevant for this section of this paper.

1. For the “FIRST 2 YEARS LIFE” section of the paper (Medline and Cochrane):

(“infant”[Title] OR “breastfeeding”[Title] OR “complementary feeding”[Title]) AND (“obesity”[Title] OR “overweight”[Title] OR “metabolic syndrome”[Title])

- with limits to “Full text” and from 2011 to 2020, March

This search revealed 312 results, out of these 22 were relevant for this section of this paper.

1. For the “DIETARY PATTERNS” section of the paper:
   1. MEDITERRANEAN DIET (Medline and Cochrane):

(“children”[Title] OR “childhood”[Title] OR “adolescent”[Title]) AND (“mediterranean diet”[Title] OR “mediterranean dietary pattern”[Title]) AND (“obesity”[Title] OR “overweight”[Title] OR “metabolic syndrome”[Title])

- with limits to “Full text” and from 2011 to 2020, March

This search revealed 247 results, out of these 3 were relevant for this section of this paper.

- 1. NORDIC DIET (Medline and Cochrane):

(“children”[Title] OR “childhood”[Title] OR “adolescent”[Title]) AND (“nordic diet”[Title] OR “nordic dietary pattern”[Title]) AND (“obesity”[Title] OR “overweight”[Title] OR “metabolic syndrome”[Title])

- with limits to “Full text” and from 2011 to 2020, March

This search revealed 24 results, out of these 3 were relevant for this section of this paper.

- 1. VEGETARIAN DIET (Medline and Cochrane):

(“children”[Title] OR “childhood”[Title] OR “adolescent”[Title]) AND (“vegetarian diet”[Title] OR “vegetarian diet pattern”[Title]) AND (“obesity”[Title] OR “overweight”[Title] OR “metabolic syndrome”[Title])

- with limits to “Full text” and from 2011 to 2020, March

This search revealed 47 results, out of these 10 were relevant for this section of this paper.

- 1. SUGAR SWEETENED BEVERAGES (Medline and Cochrane):

(“children”[Title] OR “childhood”[Title] OR “adolescent”[Title]) AND (“sugar sweetened beverages”[Title] OR “SSB”[Title] OR “SSBs”[Title]) AND (“obesity”[Title] OR “overweight”[Title] OR “metabolic syndrome”[Title])

- with limits to “Full text” and from 2011 to 2020, March

This search revealed 829 results, out of these 5 were relevant for this section of this paper.

1. For the “DIETARY MODULATION OF GUT MICROBIOTA” section of the paper (Medline and Cochrane):

(“children”[Title] OR “childhood”[Title] OR “adolescent”[Title]) AND (“gut microbiota”[Title] OR “probiotic”[Title] OR “probiotics”[Title] OR “prebiotic”[Title] OR “prebiotics”[Title] OR “synbiotic”[Title] OR “synbiotics”[Title]) AND (“obesity”[Title] OR “overweight”[Title] OR “metabolic syndrome”[Title])

- with limits to “Full text” and from 2011 to 2020, March

This search revealed 24 results, out of these 12 were relevant for this section of this paper.

1. For the “EATING BEHAVIOUR” section of the paper:
   1. SKIPPING BREAKFAST (Medline and Cochrane):

(“children”[Title] OR “childhood”[Title] OR “adolescent”[Title]) AND (“skipping breakfast”[Title] OR “breakfast”[Title] OR “skippers”[Title]) AND (“obesity”[Title] OR “overweight”[Title] OR “metabolic syndrome”[Title])

- with limits to “Full text” and from 2011 to 2020, March

This search revealed 24 results, out of these 3 were relevant for this section of this paper.

- 1. FAMILY DINNER (Medline and Cochrane):

(“children”[Title] OR “childhood”[Title] OR “adolescent”[Title]) AND (“family dinner”[Title/Abstract] OR “dinner”[Title/Abstract]) AND (“obesity”[Title] OR “overweight”[Title] OR “metabolic syndrome”[Title])

- with limits to “Full text” and from 2011 to 2020, March

This search revealed 21 results, out of these 4 were relevant for this section of this paper.

1. For the “MEAL FREQUENCY, COMPOSITION AND PORTION SIZE” section of the paper:
   1. EATING FREQUENCY (Medline and Cochrane):

(“children”[Title] OR “childhood”[Title] OR “adolescent”[Title]) AND (“eating frequency”[Title/Abstract] OR “meals frequency”[Title/Abstract] OR “meal frequency”[Title/Abstract] OR “daily meals”[Title/Abstract]) AND (“obesity”[Title] OR “overweight”[Title] OR “metabolic syndrome”[Title])

- with limits to “Full text” and from 2011 to 2020, March

This search revealed 58 results, out of these 7 were relevant for this section of this paper.

- 1. MEAL CONSUMPTION: FAST FOODS AND SNACKS (Medline and Cochrane):

(“children”[Title] OR “childhood”[Title] OR “adolescent”[Title]) AND (“fast food”[Title] OR “fast foods”[Title] OR “snacks”[Title] OR “snacking”[Title]) AND (“obesity”[Title] OR “overweight”[Title] OR “metabolic syndrome”[Title])

- with limits to “Full text” and from 2011 to 2020, March

This search revealed 39 results, out of these 11 were relevant for this section of this paper.

- 1. PORTION SIZE (Medline and Cochrane):

(“children”[Title] OR “childhood”[Title] OR “adolescent”[Title]) AND (“food portion”[Title/Abstract] OR “meals portion”[Title/Abstract] OR “portioning”[Title/Abstract] OR “portion size”[Title/Abstract]) AND (“obesity”[Title] OR “overweight”[Title] OR “metabolic syndrome”[Title])

- with limits to “Full text” and from 2011 to 2020, March

This search revealed 16 results, out of these 3 were relevant for this section of this paper.

1. For the “PHYSICAL ACTIVITY AND SEDENTARY BEHAVIOUR” (Medline and Cochrane):

(“children”[Title] OR “childhood”[Title] OR “adolescent”[Title]) AND (“physical activity”[Title] OR “sedentary time”[Title] OR “screen time”[Title]) AND (“obesity”[Title] OR “overweight”[Title] OR “metabolic syndrome”[Title])

- with limits to “Full text” and from 2011 to 2020, March

This search revealed 296 results, out of these 17 were relevant for this section of this paper.
